# Current advances on the therapeutic potential of scutellarin: an updated review

**DOI:** 10.1007/s13659-024-00441-3

**Published:** 2024-03-04

**Authors:** Yifei Xie, Guotong Sun, Yue Tao, Wen Zhang, Shiying Yang, Li Zhang, Yang Lu, Guanhua Du

**Affiliations:** 1 Beijing City Key Laboratory of Drug Target and Screening Research, National Center for Pharmaceutical Screening, Institute of Materia Medica, Chinese Academy of Medical Sciences, and Peking Union Medical College, Beijing, 100050 China; 2 Beijing City Key Laboratory of Polymorphic Drugs, Center of Pharmaceutical Polymorphs, Institute of Materia Medica, Chinese Academy of Medical Sciences and Peking Union Medical College, Beijing, 100050 China; 3 Pharmaceutical College of Henan University, Kaifeng, 475004 China

**Keywords:** Scutellarin, Pharmacological action, Experimental study, Model, Mechanism

## Abstract

Scutellarin is widely distributed in *Scutellaria baicalensis*, family Labiatae, and *Calendula officinalis*, family Asteraceae, and belongs to flavonoids. Scutellarin has a wide range of pharmacological activities, it is widely used in the treatment of cerebral infarction, angina pectoris, cerebral thrombosis, coronary heart disease, and other diseases. It is a natural product with great research and development prospects. In recent years, with in-depth research, researchers have found that wild scutellarin also has good therapeutic effects in anti-tumor, anti-inflammatory, anti-oxidation, anti-virus, treatment of metabolic diseases, and protection of kidney. The cancer treatment involves glioma, breast cancer, lung cancer, renal cancer, colon cancer, and so on. In this paper, the sources, pharmacological effects, in vivo and in vitro models of scutellarin were summarized in recent years, and the current research status and future direction of scutellarin were analyzed.

**Graphical Abstract:**

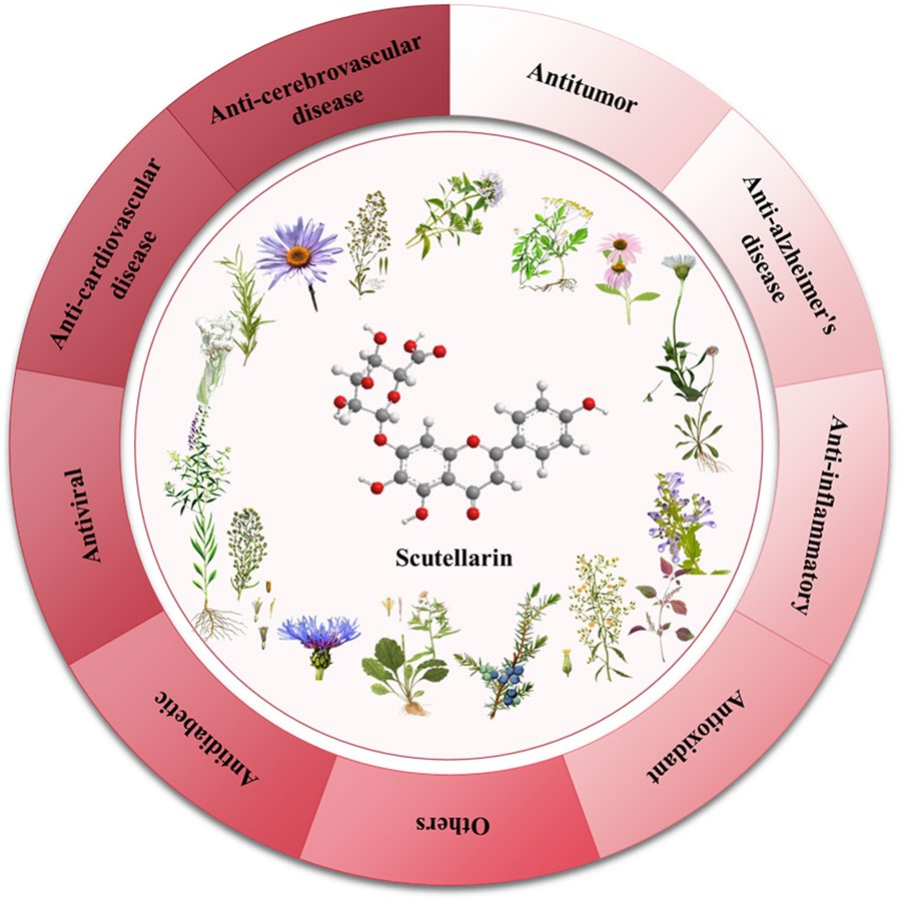

## Introduction

**Fig. 1:**
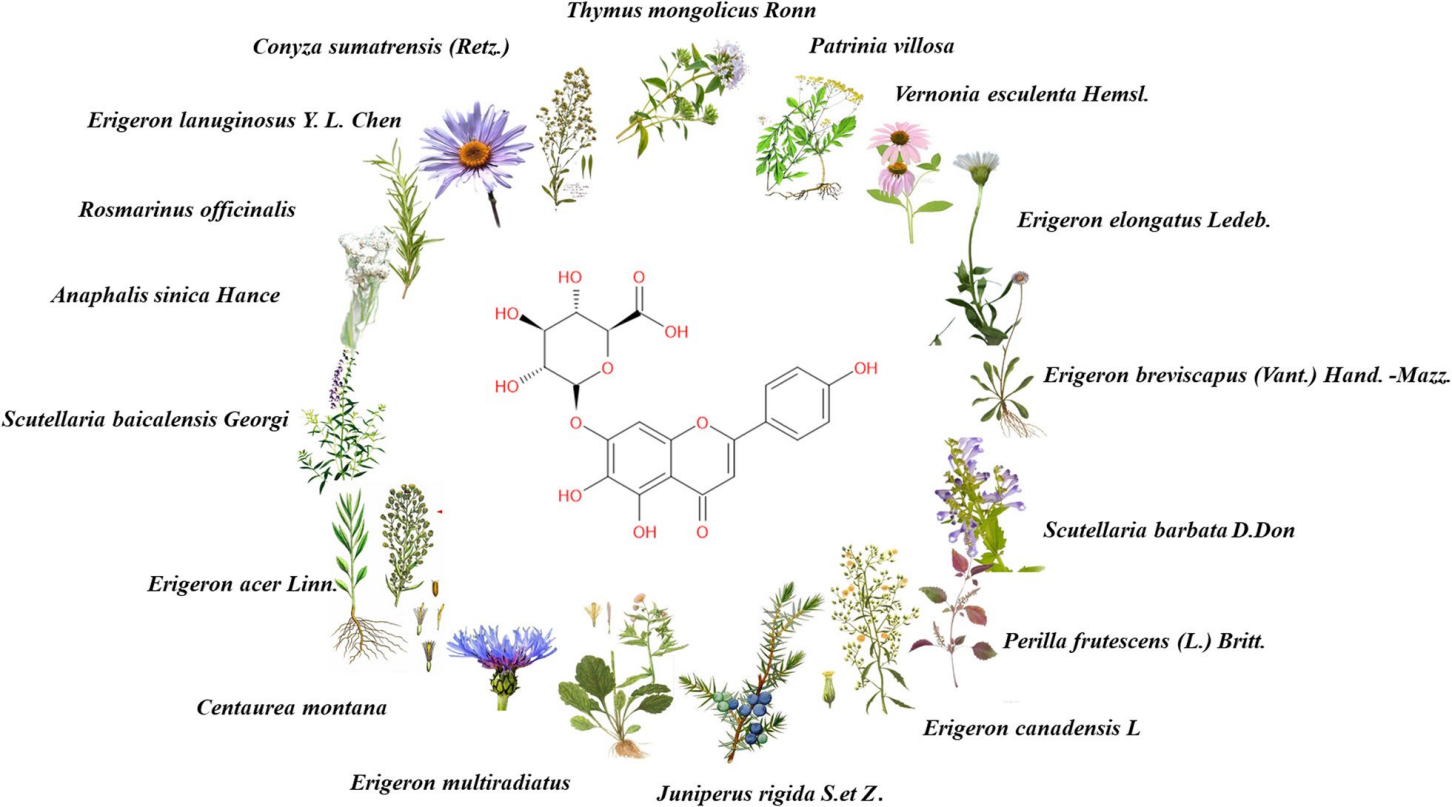
Structure and source of scutellarin

Scutellarin is a natural flavonoid extracted from plants that can be obtained from a variety of natural plants. Scutellarin is the main active substance, not only in Erigeron plants [1], but also widely distributed in Scutellaria plants [2], Opuntia plants [3], Centaurus plants [4], and Anaphalis plants [5]. Cumulative data show that scutellarin can be isolated from a variety of plants, such as *Conya sumatrensis* Retz [3], *Centaurea montana* [4], *Anaphalis sinica* Hance. [5], *Vernonia esculenta* Hemsl. [6], *Erigeron breviscapus* (Vant.) Hand.-Mazz. [7], *Scutellaria barbata* D. Don. [8], *Scutellaria baicalensis* Georgi. [9], *Erigeron multiradiatus* [10], *Conyza canadensis* L. [11], *Thymus mongolicus* (Ronniger) Ronn. [12], *Perilla frutescens* (L.) Britt. [13], *Rosmarinus officinalis* L. [14], *Juniperus rigida* S. et Z. [15], *Patrinia villosa* [16] etc. The structure, source, and function of scutellarin are shown in Fig. 1 and Table 1.

**Table 1:** Distribution of scutellarin

Latin name	Scutellarin content (%)	Extraction site	Extract method	References
*Conyza sumatrensis* (Retz.)	+	Dried whole herb	95% Ethanol	[3]
*Centaurea calcitrapa*	+	Roots and seeds	Ethanol	[4]
*Anaphalis sinica* Hance.	+	Dried whole herb	Ethanol	[5]
*Vernonia esculenta* Hemsl.	+	Dry root	Ethanol	[6]
*Erigeron breviscapus* (Vant.) Hand. –Mazz.	0.72–2.4	Dried whole herb	80% Ethanol	[7]
*Scutellaria barbata* D. Don.	0.21–0.33	Dried whole herb	80% Ethanol	[8]
*Scutellaria baicalensis* Georgi	1.690	Dry root	70% Ethanol	[9]
*Erigeron multiradiatus*	1.1	Dried whole herb	80% Ethanol	[10]
*Erigeron elongatus* Ledeb.	1.24	Dried whole herb	80% Ethanol	[10]
*Erigeron lanuginosus* Y. L. Chen.	0.78	Dried whole herb	80% Ethanol	[10]
*Erigeron acer* Linn.	1.52	Dried whole herb	80% Ethanol	[10]
*Erigeron canadensis* L.	3.2–3.4	Dried whole herb	95% Ethanol	[11]
*Thymus mongolicus* (Ronniger) Ronn.	+	Dried whole herb and above-ground part	80% Ethanol	[12]
*Perilla frutescens* (L.) Britt.	+	Roots, stems, leaves and fruits	80% Ethanol	[13]
*Rosmarinus officinalis*	+	Dried whole herb	65% Ethanol	[14]
*Juniperus rigida* S.et Z.	+	Branches and cones	70% Ethanol	[15]
*Patrinia villosa*	+	Dried whole herb	60% Ethanol	[16]

“+” indicates unknown content

Toxicological studies have proved that scutellarin is a slightly toxic or non-toxic natural small-molecule flavone compound, and the safe dose of LD50 value is 10 g/kg [17]. Based on the advantages of low toxicity and a wide range of sources, researchers found that scutellarin has a variety of pharmacological activities, including cardiovascular [18] and cerebrovascular diseases [19, 20], treatment of a variety of cancer therapies [21], and anti-inflammatory effects. In recent years, scutellarin played an important role in the treatment and protective effects of liver and kidney function caused by diabetes, the damage of brain, heart, liver, and kidney organs caused by alcohol, and eye diseases.

Animal models and cell models are important pharmacological research methods to evaluate drug activity and explore drug mechanisms, which usually need to be comprehensively evaluated by in vitro and in vivo methods combined with various models. Based on the study of different pharmacological activities of scutellarin in recent years, we combined the mechanism of scutellarin with in vivo and in vitro models to update the research progress of scutellarin’s pharmacological effects and look for changes and breakthroughs in recent years.

## Pharmacological effects

### Anti-cerebrovascular disease effect

Scutellarin is widely used in the clinical treatment of cerebral ischemia because of its anti-inflammatory, antioxidant, and vasodilator effects. The key feature of cerebral ischemia is that microglia in the brain evolve from a quiescent, branching morphology to an activated, unbranched, amoeboid morphology, which releases a large number of inflammatory mediators such as tumor necrosis factor-alpha (TNF-α), inter-leukin-1 beta (IL-1β), inducible nitric oxide synthase (iNOS), and other inflammatory mediators. TNF-α, IL-1β, iNOS, and reactive oxygen species (ROS), thereby exacerbating local inflammation and neuronal damage [22]. Chen et al. [23] found that scutellarin inhibited phosphorylated c-Jun N-terminal kinase (p-JNK), peripheral blood phosphorylated p38 mitogen-activated protein kinase (p-p38), and down-regulated inflammation in microglia by modulating the mitogen-activated protein kinases (MAPKs) pathway through the establishment of a permanent cerebral arterial occlusion model (MCAO). In addition, it can also significantly upregulate the expression level of extracellular signal-regulated kinase1/2 (p-ERK1/2), which can play a neuroprotective role. NIU [24] and others simulated neonatal hypoxic-ischemic disease (HIE) by culturing primary rat cerebral cortical neurons under the condition of hypoxia and glucose deficiency. After administration of scutellarin, it was found that scutellarin could inhibit neuronal death and regulate the Growth-associated protein (GAP43)-dependent pathway to promote axonal elongation of neurons, thus alleviating long-term nerve injury caused by HIE. Another study found that scutellarin can activate G-cyclase (GC) and produce cyclic guanosine monophosphate (cGMP) through endothelial-derived nitric oxide (NO), thus regulating the cGMP-dependent protein kinase (PKG) pathway [25–27]. PKG is a powerful vascular tone regulator. Activated PKG induces phosphorylation of vasodilator-stimulated phosphoprotein (Vasp), which in turn activates downstream ion channels and triggers endothelium-dependent vasodilation [28, 29]. DENG et al. found that scutellarin can repair oxidative stress injury in mice models of cerebral ischemia–reperfusion injury by down-regulating tMCAO-induced mRNA and protein expression of AR and NOX [30]. MENG et al. found that scutellarin can treat cerebral ischemia by regulating MARK, phosphatidylinositol 3 kinase (PI3K), hypoxia-inducible factor-1 (HIF-1), and other pathways, thus exerting vasorelaxation, anti-inflammatory and antioxidant effects [31]. Jia et al. [32] found through network pharmacology and experimental validation that Scutellarin exerts therapeutic effects on cerebral ischemia by activating astrocyte JAK2/STAT3 signaling, which provides a solid experimental basis for its clinical application. Xie et al. [33] found that Scutellarin exhibited antioxidant, anti-inflammatory, and neuroprotective effects in cerebral ischemia/reperfusion injury (CIRI) through PI3K/Akt pathway-mediated Nrf2 activation. Zhang et al. [34] found that scutellarin inhibits the inflammatory process through nuclear factor κ-B p65 and p38 mitogen-activated protein kinase signaling pathways and protects against brain damage in ischemically injured rats. Wang et al. [35] found that scutellarin exerts an effective effect on acute-phase ischemic brain injury by modulating neurotransmitter activity and reducing the toxicity of excitatory amino acids in neurons. M2 Microglia can exert neuroprotective effects and promote tissue repair. Chen et al. [36] found that scutellarin may directly promote the polarization of M2 microglia and their expression of neurotrophic factors. The protective effect was exerted through the inhibition of JNK and p38 signaling pathways. In addition, lucigenin promotes the polarization of M2 microglia by enhancing the ERK1/2 signaling pathway. In addition, scutellarin may also alleviate acute alcoholic brain injury by stimulating the activity of antioxidant enzymes and inhibiting the expression of syndromic factors [37]. Types of action and models of scutellarin in anti-cerebrovascular disease are showed in Table 2.

**Table 2:** Types of action and models of scutellarin in anti-cerebrovascular disease

Disease model	Dose	Animal/cell	Weight/g	In vivo/in vitro	References
Cerebral artery occlusion model(MCAO)	100 mg/kg	Male SD rats	250–280 g	In vivo	[23]
	100 mg/kg	Male SD rats	250–280 g	In vivo	[32]
Cerebral artery occlusion reperfusion model (MCAO/R) OGD/R model	20 mg/kg (rats) 25, 50, 100 μM (cell)	Male SD rats HT22 cell line	270 ± 10 g	In vivo In vitro	[33]
Cerebral artery occlusion reperfusion model (MCAO/R)	6, 12 mg/kg	SD rats	–	In vivo	[35]
Middle cerebral artery (MCAO)	40, 80 mg/kg	Male SD rats	200–220 g	In vivo	[34]
Cerebral artery occlusion (MCAO) LPS-stimulated BV-2 cell model	100 mg/kg (rats) 0.54 μM (cell)	Adult male SD rats BV-2 microglia	150 ± 10 g	In vivo In vitro	[36]
Hypoxic-ischemic encephalopathy model (HIE)	20 mg/kg 0.1–100 μmol	Timed pregnant female SD rat primary cortical neuronal cells	–	In vitro	[24]
Cerebral ischemia–reperfusion model (IR)	45, 90 mg/kg	SD rats	–	In vivo	[29]
Transient middle cerebral artery occlusion (tMCAO)	50, 100 mg/kg	Male C57BL/6N mice	20–24 g	In vivo	[30]
Acute alcohol mice brain injury model	10, 25, 50 mg/kg	BALB/c male mice	18‒22 g	In vivo	[37]

### Anti-cardiovascular disease effect

Scutellarin has pharmacological effects such as slowing down heart rate, regulating myocardial contractility, dilating blood vessels, reducing cardiac preload and afterload, dilating coronary arteries, and increasing myocardial oxygen supply. It has been widely used in the clinical treatment of cardiovascular diseases. Li et al. [38] found that scutellarin mediates I/R-induced cardiomyocyte apoptosis and cardiac dysfunction by regulating the activation of Bcl-2/Bax/Caspase-3 signaling pathway, so as to improve treatment and improve ischemic heart disease. Zhou et al. [39] found that scutellarin ameliorates I/R-induced cardiomyocyte apoptosis and cardiac dysfunction by activating the Bcl-2/Bax/Caspase-3 signaling pathway via the cGAS-STING signaling pathway. Wang et al. [40] found that scutellarin protects against myocardial ischemia–reperfusion injury EPK1/2-CREB regulates mitochondrial autophagy. Fu et al. [41] found that scutellarin significantly reduced lipid levels and increased antioxidant enzymes in atherosclerotic (AS) rats by inhibiting mammalian sterile20-like kinases 1 (Mst1) phosphorylation, Yes-associated protein (YAP) phosphorylation, phosphorylation of forkhead box O3A (FOXO3a), nuclear translocation of FOXO3a, and up-regulation of protein kinase Bm (AKT) expression. Regulates downstream genes to inhibit vascular endothelial cell injury and apoptosis, thus exerting anti-AS effects. Scutellarin has also been used in the treatment of diabetic cardiomyopathy, where it can lower blood glucose, total cholesterol, triglyceride, and LDL levels, up-regulate HDL levels, down-regulate the levels of lactic dehydrogenase 1 (LDH1) and creatine kinase (CK), and promote mRNA and protein expression of autophagy-related factors Beclin-1 and Lc3-II, and inhibit the apoptosis-related factors cysteine aspartic acid-specific protease (caspase), as well as the B-cell lymphocyte-associated X protein (BAX). Protein (BAX), and Cytochrome C (Cyt-C) mRNA and protein expression, thereby up-regulating autophagy-associated factors to promote autophagy signaling pathway and down-regulating apoptosis-associated factors to inhibit apoptosis signaling pathway, which improves the cardiac morphology and reduces apoptosis of cardiac myocytes and serves as a treatment for type 2 diabetic cardiomyopathy [42]. Huo and others [43] found that scutellarin could improve oxidative stress, inflammation, and reduce apoptosis by modulating NRF2/KEAP/ARE, TLR4/MYD88/NF-κB, and apoptosis pathways to treat and prevent myocardial injury complicated by type 2 diabetes mellitus. Qu et al. [44] used isolated rat hearts to study the effect of scutellarin on acute myocardial ischemia/reperfusion injury and found that scutellarin significantly increased the expression I/R (ischemia/reperfusion)-induced decrease in PPARγ and Nrf2 protein, and decreased the I/R-induced elevation of NF-κB protein expression to achieve cardioprotection. scutellarin- scutellarin (PAE) polylactic acid-glycidylglycolic acid (PLGA) nanoparticles (NPs) made by combining scutellarin with PLGA were able to reduce CK, LDH, and AST levels in serum, decrease apoptosis, and improve cardiac function [45]. Types and models of scutellarin action in anti-cardiovascular diseases are shown in Table 3.

**Table 3:** Types of action and models of scutellarin in anti-cardiovascular disease

Disease model	Dose	Animal/cell	Weight/g	In vivo/in vitro	References
Ischemia/reperfusion (I/R)	20 mg/kg	Male C57BL/6 mice	18‑25 g	in vivo	[38]
DOX-induced cytotoxicity in H9c2 cells, CFs, and HUVECs	6, 12, 25, 50, 100 μM	Rat H9c2 cells, CFs, and HUVECs	–	In vitro	[39]
Myocardial ischemia–reperfusion animal model (MIR) Myocardial ischemia–reperfusion injury cell model	50 mg/kg (mice) 10, 30, 50, 100 μM (cell)	Male C57BL/6 mice H9c2 cell	12 weeks of age	In vivo In vitro	[40]
atherosclerosis model (AS)	6.25, 25 mg/kg	Male SD rats	200–220 g	In vivo	[41]
Diabetes complicated cardiomyopathy model (T2DC)	100, 200 mg/kg	Male SD rats	180–200 g	In vivo	[42]
	10, 20 mg/kg	Swiss mice	22 ± 3 g	In vivo	[43]
Isolated Langendorff rat heart model	1, 5, and 25 µg/ml	Male SD rats	250–300 g	In vitro	[44]
rat myocardial ischemia (MI)	5 mg/kg	Male SD rats	250 ± 20 g	In vivo	[45]

### Antitumor effect

**Fig. 2:**
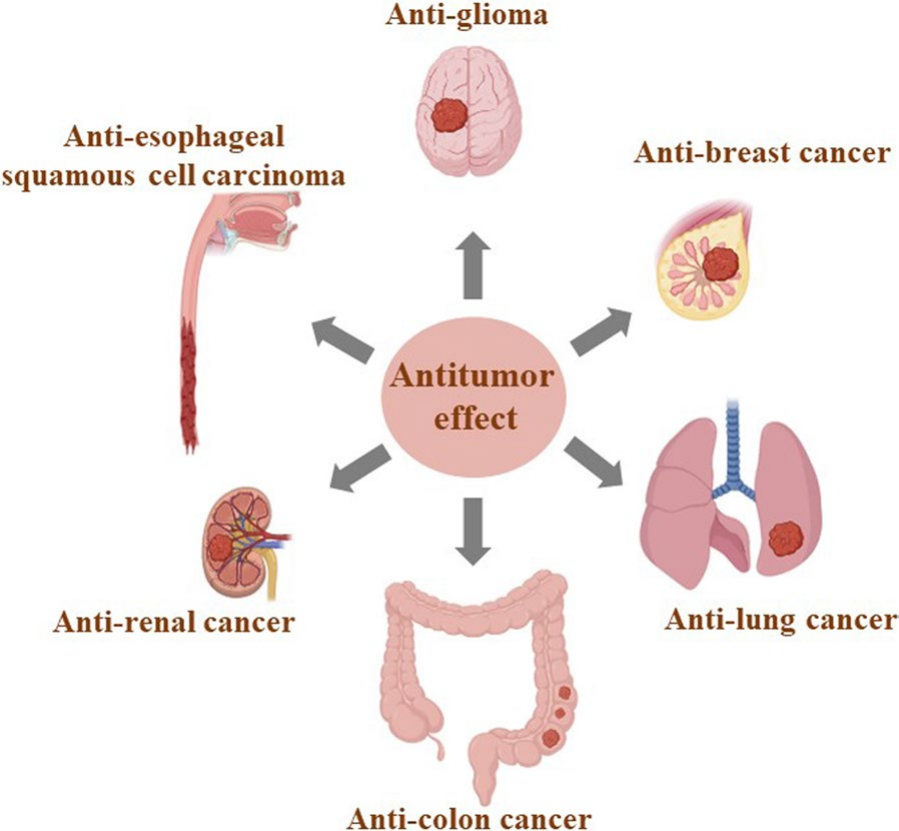
Categories of human cancers that can be prevented and treated by scutellarin

In vivo and in vitro studies have shown that scutellarin can be used for the prevention and treatment of a variety of human cancers, and the types of cancers are shown in Fig. 2. Types of action and models of scutellarin in antitumor effects are shown in Table 4.

**Table 4:** Types of action and models of scutellarin in anti-tumor effect

Type of disease	Disease model	Dose	Animal/cell	Weight/g	Positive control	Dose	In vivo/in vitro	References
Anti-colon cancer	Oxidized azo-induced colon cancer	25, 50, 100 mg/kg (mice) 60–400 μmol (cell)	Male C57BL/6 mice HT-29 cell	–	–	–	In vivo In vitro	[46]
	Human colorectal cancer (CRC) cell lines SW620 xenograft human tumor model in mice	6.25, 12.5, 25, 100, 160, 200, 300, 400 μg/mL 6.4, 12.8, 25.6 mg/kg	Human SW620, Caco2, HT29 cells male nude mice	– 25 g	–	–	In vitro In vivo	[47]
	Colitis-associated colorectal cancer (CAC) model	25, 50, 100 mg/kg 80, 160, 320 μM	Male C57BL/6 mice IEC-6 rat small in testine epithelial cells	6 weeks old	GANT61	20 mg/kg	In vivo In vitro	[48]
Anti-renal cancer	Xenograft mouse model renal cell carcinoma	30, 60 mg/kg 30, 60, 90 μM	Male BALB/c nude mice ACHN,786-O cell	5 weeks old	–	–	In vivo In vitro	[49]
Anti-lung cancer	Lung cancer	100, 250, 500 μM	A549 cell	–	–	–	In vitro	[54]
	A549 xenograft nude mouse model Non-small cell lung cancer (NSCLC)	0–500 μg/mL	Male BALB/c nude mice A549 cell, H1975 cell	4 weeks old	–	–	In vitro	[55]
Anti-glioma cancer	Glioma in situ in rats	20, 50 mg/kg	Female SD rats	–	–	–	In vivo	[56]
	Glioma cell	10, 20, 40, 80, 160 μg/mL	Glioma cell line T98G	–	–	–	In vitro	[57]
	Glioma cell	10 mg/mL	U251, M059K, and SF-295 cell lines	–	5-fluorouracil	10 mg/mL	In vitro	[58]
Anti-breast cancer	Triple-negative breast cancer (TNBC) model	10 mg/kg	Female Balb/c mice	4 weeks old	–	–	–	[59]
		1, 10 mg/kg 5, 20 μM	Female nude Balb/c mice female Balb/c mice HMMECs HUVECs	4 weeks old	–	–	In vivo In vitro	[60]
Anti-esophageal squamous cell carcinoma (ESCC)	Patient-derived xenograft esophageal squamous carcinoma (ESCC) esophageal squamous cell carcinoma (ESCC) model	10 mg/kg 2, 5, 10 μM	Female mice KYSE cell	6–9 weeks old	–	–	In vivo In vitro	[61]
Anti-pancreatic cancer	Pancreatic cancer (PaCa) cell model	100 μM	Human PaCa cell lines	–	–	–	In vitro	[62]
Anti-hepatocellular carcinoma	Pancreatic cancer cell model	2.5, 5, 10, 20, 40, 80, 100 µmol/L	Mouse HCC cell line, Hepa1-6	–	–	–	In vitro	[63]

#### Anti-colon cancer effect

Zeng et al. [46] found that scutellarin reduced the expression levels of TNF-α and TNF-1β in mouse serum, up-regulated the expression of BAX, and down-regulated the expression level of Bcl-2 in mouse cancer tissues through down-regulation of the Wnt/β-Catenin signaling pathway, and then inhibited the proliferation and migration of HT-29 cancer cells, and induced cell apoptosis. Scutellarin is one of the active ingredients of *Erigeron breviscapu* (Dengzhanxixin in China) injection (EBI). By activating the necrotic RIPK3/MLKL classical signaling pathway, EBI greatly promoted intracellular ROS production, significantly inhibited the proliferation of three human colorectal cancer (CRC) cell lines, and effectively inhibited the migration and invasion of SW620 cells, thus exerting anti-tumor effects. In addition, in the SW620 xenograft mouse model, EBI significantly inhibited tumor growth and lung metastasis and effectively circumvented drug-resistant apoptosis providing a new therapeutic pathway for anti-colon cancer [47]. Scutellarin significantly ameliorated AOM/DSS-induced CAC in mice and induced apoptosis in CAC tissues by inhibiting NF κB (nuclear factor κB)-mediated inflammation and the Hedgehog signaling axis. In addition, scutellarin inhibited cell proliferation, migration, and colony formation, and induced apoptosis in SW480 cells by down-regulating the Hedgehog signaling pathway activity, and reduced NFκB-mediated inflammatory responses in TNF-α-stimulated IEC-6 cells [48].

#### Anti-renal cancer effect

Deng et al. [49] found that scutellarin enhanced the expression of phosphatase and tensin homologue (PTEN) protein, a bispecific protein phosphatase identified as a tumor suppressor in a variety of human malignant tumors, and that activated PTEN caused second messenger lipid phosphatidylinositol 3,4,5-trisphosphate (PIP3) dephosphorylates, thereby contributing to its counteracting phosphatidylinositol-3-kinase (P13K) activity and AKT phosphorylation, inhibiting the PI3K/AKT pathway, which plays an important role in tumor cell proliferation, angiogenesis, and survival, and induces apoptosis of tumor cells, stalling their cell cycle at the G0/G1 phase, thus inhibiting the proliferation and invasion of renal cancer cells, suggesting that scutellarin has the potential to be a potential drug for the treatment of renal cancer.

#### Anti-lung cancer effect

TGF-β is considered a tumor suppressor due to its strong growth inhibitory activity in a wide range of cells. The TGF-β/Smad signaling pathway plays an important role in tumor cell proliferation, differentiation, and apoptosis. However, scutellarin modulates this pathway to induce apoptosis in tumor cells, and it has been reported that scutellarin selectively reduces the survival rate of tumor cells without affecting normal cells [50–53]. He and others [54] found that scutellarin could enhance ^125^I-induced apoptosis and the antiproliferative effect of tumor cells through down-regulation of the AKT/mTOR pathway, which provided the possibility of its combination therapy. Sun and others [55] found that scutellarin could promote caspase-3-dependent apoptosis induced by the first-line antitumor drug cisplatin via the ERK/P53 pathway and promote cisplatin-induced cytotoxicity autophagy via the c-Met-AKT pathway, which in turn acted to reverse the unique properties of cisplatin resistance.

#### Anti-glioma cancer effect

Wang et al. [56] found that scutellarin was able to inhibit the growth of glioma, as well as the proliferation and migration of glioma cells. In addition, scutellarin could significantly reduce the expression of baculovirus inhibitor of apoptosis protein 5 (BIRC5), thereby reversing the inhibition of apoptosis in glioma cells and exerting its anti-glioma effect. Therefore, Scutellarin may become a new potential targeted drug for the treatment of gliomas. Du et al. [57] found that Scutellarin inhibited the proliferation of glioma cells by upregulating miR-15a expression. Scutellarin-induced apoptosis and cell cycle in the G2/M phase SF-295 cell line inhibited cell proliferation in a dose-dependent manner and inhibited the growth of gliomas through the p63 signaling pathway in a dose-dependent manner, which was similar but weaker than the effect of 5-fluorouracil [58].

#### Anti-breast cancer effect

Triple-negative breast cancer (TNBC) is an aggressive breast cancer subtype with high blood vessels and frequent metastasis. Scutellarin blocks the interaction between TNF-α and TNFR2, and inhibits the activation of RUNX1 and the production of G-CSF in endothelial cells associated with TNBC downstream, thus reducing the metastasis of TNBC [59]. Scutellarin reduces the tendency of decreased connexin expression by regulating the TNFR2-ERK1/2-EZH2 signaling pathway, which inhibits TNF-α initiated vascular endothelial barrier disruption and thus reduces TNBC metastasis [60].

#### Treatment of other tumors

AKT is a serine/threonine kinase, belonging to the AGC family, which can regulate cell proliferation and survival, and scutellarin can activate this pathway, causing tumor cells to arrest with the G2 phase, which plays a role in the treatment of esophageal squamous carcinoma [61]. Girdin is an actin-binding protein, that is involved in cancer invasion and angiogenesis, and is a prognostic biomarker. Girdin can participate in pancreatic cancer (PaCa) migration mediated by EGF signal, and SCU can inhibit cancer invasion by inhibiting Girdin, thus playing a role in anti-cancer [62]. Li et al. used an aminoethyl anisamide-targeted copolymerization approach to amplify the delivery capacity of scutellarin to facilitate the in vivo application of scutellarin in hepatocellular carcinoma (HCC) immunotherapy and demonstrated that scutellarin has the potential to trigger immunogenic cell death (ICD) in hepatocellular carcinoma HCC [63] ).

### Anti-diabetic effect

#### Therapeutic effect on insulin resistance

Insulin resistance (IR) is a condition in which the peripheral tissues of the body become less sensitive to insulin and is present in various metabolic disorders such as diabetes mellitus, obesity, and hypertension. IR can occur in the liver, muscle, and adipose tissues, and when the levels of insulin, free fatty acids, or glucose in them are elevated tissues produce ROS and oxidative stress, as a vicious cycle IR and oxidative stress reinforce each other and exacerbate the body damage. LUAN et al. found that scutellarin reduced insulin-dependent lipid accumulation and mRNA expression upregulated Akt phosphorylation and improved the insulin signaling pathway in HepG2 cells in vitro. Down-regulation of rapamycin-targeted protein (mTOR) phosphorylation and n-SREBP-1c protein levels in high-fat diet (HFD)-fed mice and reduction of lipid accumulation in IR and lipid metabolism disorders through mTOR-dependent pathways [64]. Gao et al. found that scutellarin could activate the AMPK-α-mediated insulin signaling pathway, which in turn up-regulated P85α, activated the PI3K/AKT pathway, and ultimately affected the expression of the glucose transporter GLUT4, which indirectly exerted a hypoglycemic and lipid-lowering effect. in addition, scutellarin could also be a multi-targeted treatment for IR through its anti-oxidative stress, but the mechanism of the synergism of scutellarin’s modulation of the AMPK pathway and the anti-oxidative stress in the treatment of IR is still unclear and needs to be further investigated [65].

#### Antidiabetic-induced retinal injury

Scutellarin has a favorable therapeutic effect on diabetes-induced endothelial cell damage and retinopathy (DR). Scutellarin reduces the expression of BCL2, BAX, and fine cYTC, and inhibits apoptosis through a mitochondria-dependent pathway to treat diabetes-induced endothelial cell damage. Scutellarin also improves the decrease in mitochondrial membrane potential with ROS overload, decreases the protein expression of superoxide dismutase (SOD), and promotes the expression of microtubule-associated protein LC3II and autophagy-related gene ATG5. In addition, scutellarin can upregulate mitogen phagocytosis by modulating the PINK1/Parkin signaling pathway, thereby exerting a protective effect against hyperglycemia-induced vascular endothelial cell injury [66]. Vascular endothelial growth factor (VEGF) is the main cause of retinal neovascularization, vascular leakage, and is a therapeutic target for anti-angiogenesis in DR. Oral administration of scutellarin attenuates microvascular dysfunction caused by hyperglycemia and hypoxia in vitro and in vivo and inhibits vascular neovascularization, which may be related to its inhibition of VEGF and its downstream proteins, p-ERK, phosphorylated adhesion plaque kinase (p-FAK), and phosphorylated tyrosine protein kinase (p-Src) activation. It is evident that scutellarin is a VEGF inhibitor and has the potential to be a therapeutic agent for diabetic microangiopathy. Li et al. [67] found induced DR cell focussing in DM rats, particularly in retinal ganglion cells (RGCs), and found that scutellarin administration significantly inhibited cell focussing in DR and explained the molecular network mechanisms involved.

#### *Antidiabetic-induced liver and kidney damage*

Fan et al. [68] found that scutellarin may ameliorate T2DM liver injury by inhibiting hepatocyte apoptosis in vitro and in vivo. Huang et al. [69] found that scutellarin was effective in ameliorating various features of diabetic nephropathy (DN) in vivo, including proteinuria, glomerular dilatation, accumulation of tethered matrix, renal fibrosis, and podocyte injury, by modulating the TGF-β1 signaling pathway and its interaction with the Erk and Wnt/β-linker pathways. Scutellarin plays an important role in the treatment of diabetes and its complications, and types of action and models informations are showed in Table 5.

**Table 5:** Types of action and models of scutellarin in anti-diabetic effect

Type of disease	Disease model	Dose	Animal/cell	Weight/g	Positive control	Dose	In vivo/in vitro	References
Anti-insulin resistance	High fat-diet-fed (HFD)mice	50 mg/kg	Male C57BL/6 J mice HepG2 cells	7 weeks old	–	–	In vivo	[63]
	High-fat diet (HFD) mice PA-treated HepG2 cells	50, 150 mg/kg 10, 30, 50 μM	Male C57BL/6 mice HepG2 cells	6–8 weeks old	Metform-in	200 mg/kg	In vivo In vitro	[64]
Anti-diabetic-induced retinal injury	High glucose (HG)-induced injury to human umbilical vein endothelial cells (HUVEC)	3, 10, 30 μM	HUVECs cell	–	–	–	In vitro	[66]
	Diabetic retinopathy model (DR)	50 mg/kg	Male SD rats	Adult	–	–	In vivo	[67]
Antidiabetic-induced liver damage	Type 2 Diabetes Model (T2DM) Human LO2 hepatocytes	50, 10, 200 mg/kg 0.1, 0.2, 0.4, 0.8, 1.0 mmol/L	SD rats LO2 cells	60–180 g	Rosiglita-zone	5 mg/kg	In vivo In vitro	[68]
Antidiabetic-induced kidney damage	Diabetic nephropathy (DN) mice	10, 40 mg/kg	Male C57BL/6 J mice	8 weeks old	Empaglif-lozin	20 mg/kg,	In vivo	[69]

### Therapeutic role in Alzheimer’s disease

Alzheimer’s disease (AD) is a neurodegenerative disease associated with aging, characterized by progressive memory impairment, and cognitive and social decline. At present, there is no drug to cure this disease. As for the pathogenesis of AD, it is widely assumed that amyloid (Aβ) aggregates and is accompanied by the production of neurotoxic ROS. In addition, gene mutation, abnormal phosphorylation of tau protein, neuroinflammation, cholinergic injury, and imbalance of neurovascular lesions are also causes of AD. Scutellarin binds to Aβ-42 receptors in the brain, effectively inhibits the generation of Aβ oligomers and fibers, attenuates its induced neurotoxicity and reduces the expression of phosphorylated Tau protein, increases the expression levels of superoxide dismutase and acetylcholine, inhibits the expression levels of ROS and pro-inflammatory factors TNF-α and IL-6, and attenuates neuroinflammation. It also antagonizes Aβ-induced expression of anti-apoptotic protein Bcl2, inhibits expression of pro-apoptotic protein Bax and cleaved caspase 3, reduces apoptosis, and alleviates AD [70–73]. Defective mitochondrial bioenergetics and its resulting low glucose metabolism are key pathophysiologic regulators that promote neurodegeneration, Sheng et al. [74] found that scutellarin rescue mitochondrial damage by improving mitochondrial glucose oxidation via the Pdk-Pdc axis and that active components that ameliorate mitochondrial bioenergetic deficiencies may be valuable in the treatment of neurological disorders. Microglia are essential for the development and homeostasis of the neonatal central nervous system (CNS). Types of action and models of scutellarin in the treatment of Alzheimer's diseases are showed in Table 6.

**Table 6:** Types of action and models of scutellarin in Alzheimer’s disease

Disease model	Dose	Animal/cell	Weight/g	In vivo/in vitro	References
Mouse hippocampal neuronal L‑Glu‑damaged HT22 cells	20 mg/kg 5, 15 μM	Male Balb/c mice HT22 cell line	20–24 g	In vivo In vitro	[70]
Brain Aβ amyloidosis model	50 mg/kg 2.5, 5, 10 µM	Half male and half female C57BL/6 mice SH-SY5Y cell line	Age-matching	In vivo In vitro	[71]
Rodent model of AD	50 mg/kg 0, 5, 10, 15, 30 μg/mL	Wistar rats Rat PC12 cell	200 g	In vitro In vivo	[72]
Chronic cerebral hypoperfusion induced by permanent bilateral common carotid artery occlusion (pBCAO)	10, 30 mg/kg	Male SD rats	280–300 g	In vivo	[73]
Chronic cerebral hypoperfusion (CCH) model	100 mg/kg	Male SD rats	200 ± 10 g	In vivo	[74]

### Anti-inflammatory effect

As a natural small molecule, scutellarin has good anti-inflammatory activity and can be used in the prevention and treatment of pneumonia, arthritis, and neuroinflammation. scutellarin can inhibit the inflammatory response by modulating the NF-ĸb/NLRP3 pathway, which exerts an antagonistic effect [75]. Lipopolysaccharide (LPS) is a common inflammatory factor that activates the inflammatory sensory protein caspase-11 and produces an inflammatory response. Scutellarin induces Ser/Thr phosphorylation of caspase-11 at PKA-specific sites, which counteracts the activation of caspase11 and antagonizes the inflammatory response caused by LPS [76]. Cerebrovascular diseases are often accompanied by neuroinflammation, which can lead to increased morbidity and mortality. In the central nervous system, microglia are resident innate immune cells Neuroinflammation is regulated by microglia, which play a dual role in the developing brain, both neuroprotective and neurotoxic. Activated microglia release inflammatory factors such as TNF-α, IL-1β, and NO, which further stimulate microglia activation and promote the accumulation of pro-inflammatory mediators, ultimately leading to neuronal death and exacerbating brain damage. Scutellarin can inhibit the production of inflammatory factors by regulating the AKT/NF-κB and p38/JNK pathways, which in turn inhibits microglia activation to alleviate neuroinflammation [77]. In addition, it has been shown that scutellarin can also alleviate and treat osteoarthritis by modulating the NF-ĸB pathway [78]. Yang et al. [79] found that scutellarin significantly reduced subchondral bone loss and cartilage degeneration in a mouse model of cartilage injury in medial meniscus (DMM) destabilization and in ovariectomy (OVX)-induced mouse subchondral bone loss. By inhibiting interleukin 1β-induced extracellular matrix degradation of cartilage NF-κB/mitogen-activated protein kinase (NF-κB/MAPK) signaling pathway. Li et al. [80] found that scutellarin attenuated complete Freund's adjuvant (CFA)-induced rheumatoid arthritis (RA) in mice by modulating the Keap1/Nrf2/HO-1 pathway, and the results provide preliminary evidence for the treatment of arthritis with Scutellarin. Ulcerative colitis (UC) is an inflammatory bowel disease of unknown etiology that lacks effective treatments, Aksit et al. [81] found that scutellarin may prevent UC by downregulating pro-inflammatory cytokines and inhibiting apoptosis and oxidative stress. Types of action and models of scutellarin in anti-inflammatory effects are shown in Table 7.

**Table 7:** Types of action and models of scutellarin in anti-inflammatory and antioxidant effect

Type of disease	Disease model	Dose	Animal/cell	Weight/g	Positive control	Dose	In vivo/in vitro	References
Anti-inflammatory	Idiopathic pulmonary fibrosis (IPF)	30, 60, 90 mg/kg	Male BAL/bc mice	20–30 g	–	–	In vivo	[75]
	LPS-induced depression animal model	15, 30, 45 mg/kg	Adult male SD rats	200–220 g	Fluoxetine	–	In vivo	[76]
	LPS-induced mouse model of osteoarthritis LPS-induced	50, 100 mg/kg 12.5, 25, 50 μmol/L	Female C57BL/6 mice Murine J774A.1 macrophage cell line	6–8 weeks old	–	–	In vivo In vitro	[77]
	Osteoarthritis model (OA) mice Medial meniscus (DMM) model	50 mg/kg 0, 15, 30, 60 mM	C57BL/6 mice measured chondrocyte	2 weeks old	–	–	In vivo	[78]
	DMM model mice medial meniscus (DMM) model	25, 50 mg/kg 0, 1.56, 3.12, 6.25, 12.5, 25, 50, 100 and 200 μM	female C57/BL mice Mouse ATDC5 cells	18–22 g	–	–	In vivo In vitro	[79]
	Rheumatoid arthritis model (RA)	20 mg/kg	Male C57BL/6 mice	25–30 g	Leflunomide	4 mg/kg	In vivo	[80]
	Acetic acid-induced ulcerative colitis (UC) Model	20 mg/kg	Adult male SD rats	250–320 g	Sulfasalazine TUNEL-positive cells	100 mg/kg	In vivo	[81]
Anti-oxidant	H2O2-induced oxidative damage of cells	25, 50, 100 μM	ARPE-19 cell	–	–	–	In vitro	[83]

### Antioxidant effect

The presence of phenolic hydroxyl groups in the chemical structure of scutellarin provides hydrogen atoms to exert antioxidant effects. Scutellarin can effectively scavenge a variety of free radicals such as 1,1-diphenyl-2-trinitrophenylhydrazine (DPPH), 2,2ʹ-biamine-bis-(3-ethylbenzothiazoline-6-sulfonic acid) (ABTS+), and superoxide anion (O2–), and mitigate the damage caused by the accumulation of free radicals on the body [82]. Hu et al. found that scutellarin could antagonize the effects of H_2_O_2_-induced damage to the retina in the mouse retina by culturing H_2_O_2_-induced damage to the retina in the mouse retina cells (APRE-19), and after administration of scutellarin, they found that it could antagonize the H_2_O_2_-induced increase in BAX expression and decrease in bcl-2 expression, which in turn decreased the expression of ROS, MDA, SOD, and GSH, reduced oxidative damage, and increased the survival rate of APRE-19 [83]. Types of action and models of scutellarin in antioxidan effect is shown in Table 7.

### Antiviral effect

Modern studies have shown that scutellarin has some antiviral activity. Its antiviral pharmacological action is a specific manifestation of the purgative and detoxifying action in classical Chinese medicine [84]. Details of scutellarin’s antiviral activity were reported as research progressed. Wang et al. [85] found that scutellarin significantly inhibited the activities of sortase A (SrtA) and caseinolytic peptidase P (ClpP) of methicillin-resistant *Staphylococcus aureus* (MRSA) strain USA300 by screening hundreds of compounds, fluorescence quenching assay and molecular docking results showed that scutellarin directly binds to SrtA molecules, and also inhibits hemolytic activity of Staphylococcus aureus (SA) by inhibiting Hla expression in a SrtA-independent way. It can also inhibit the hemolytic activity of SrtA by suppressing the expression of Hla in a SrtA-independent manner. Therefore, the combination of scutellarin with vancomycin has the effect of preventing MRSA invasion of A549 cells and pneumonia in mice.

### Protect the liver and kidney

Scutellarin may be a promising drug for the prevention of liver and renal injury. Miao et al. [86] established a model of CCL4-induced hepatic injury in mice and found that scutellarin exerted potential CCl4-induced hepatic injury by inhibiting the CYP2E1 and IĸBα/NF-ĸB pathways, modulating the intestinal microbiota, and endogenous metabolites involved in lipid metabolism and bile acid homeostasis, and exerting potential hepatoprotective effects. Scutellarin can play a protective role in renal protection. Dai et al. found that Scutellarin activated Nrf2 signaling protects the kidney from ischemia/reperfusion-induced oxidative damage by reducing inflammatory factors through up-regulation of the HO-1 pathway as revealed in vivo and in vitro experiments [87]. Acute renal injury (AKI) has a high mortality and morbidity. The oxidative stress induced in the kidneys after acute and excessive alcohol intake leads to acute kidney injury (AKI), resulting in causing severe swelling and damage groups of tubular epithelial cells with glomerular atrophy, necrosis, and inflammatory infiltration. Yang et al. found that langoustine exerts a protective effect against AKI through anti-inflammation and antioxidant activity [88]. Shahmohammadi et al. found that scutellarin can inhibit acute renal injury induced by regulating Nrf2/PPAR-c/PGC-1a/NF-kB/TLR4 [89].

### Other effects

Scutellarin also has pharmacological activities such as treatment of glaucoma, liver protection, kidney protection, and hyperuricemia. Zhu et al. established a mouse model of glaucoma and found that scutellarin could maintain the retinal structure and visual function of mice when intraocular pressure was elevated, suggesting that scutellarin is a potentially novel neurotherapeutic agent used in the treatment of glaucoma [90]. Li et al. found that scutellarin reduced renal injury and lowered the glomerular filtration rate in hyperuricemic mice (HN), which may be alleviated by a regulatory mechanism of extracellular cellular communication network factor 1 (CCN1) on the activation of NLRP3 inflammatory vesicles [91]. Duan et al. found that scutellarin inhibited the onset and duration of convulsions and reduced the severity of PTZ-induced seizures in mice by modulating changes in the levels of gamma-aminobutyric acid (GABA), glutamate, and dopamine, as well as the activities of Ca^2+^ ATPase and Na^+^ K^+^ ATPase [92]. Types of action and models of scutellarin in other effects are shown in Table 8.

**Table 8:** Types of action and models of scutellarin in other effects

Type of disease	Disease model	Dose	Animal/cell	Weight/g	Positive control	Dose	In vivo/in vitro	References
Anti-viral	Mice pneumonia model	Mice pneumonia model	Male and female C57BL/6 J mice	6–8 weeks old	–	–	In vitro	[85]
Protect liver injury	BALB/c mice were injected with CCl4	0.03, 0.06, and 0.12 mmol/kg	BALB/c mice	18–22 g	Bifendate	0.4 mmol/kg	In vivo	[86]
Protect renal injury	I/R-AKI model	50 mg/kg 20 μM	Male Wistar rats Human renal tubular epithelial cells (HK-2)	250–300 g	–	–	In vivo In vitro	[87]
	Alcohol-induced AKI model	10, 25, 50 mg/kg	Male BALB/c mice	18–22 g	Icariin	60 mg/kg	In vivo	[88]
	LPS-instigated model of AKI	25, 50, 100 ml/kg	C57BL/6 mice	18–22 g	–	–	In vivo	[89]
Glaucoma treatment	Injected with hydrogel into chronic IOP elevation	300 mg/kg	C57BL/6 J mice	15–18 weeks	–	–	In vivo	[90]
Anti-hyperuricemia	Hyperuricemia nephropathy (HV)	20 mg/kg	C57BL/6 mice	25–27 g	–	–	In vivo	[91]
Anti-convulsant	Pentylenetetrazol (PTZ) kindling epilepsy model	10, 20 mg/kg	Male Swiss mice	30 g	Diazepam	5 mg/kg	In vivo	[92]

## Conclusion

Scutellarin is a slightly toxic or non-toxic natural small-molecule flavone compound with good effects against cardiovascular and cerebrovascular diseases, anti-tumor, anti-insulin resistance, anti-virus, anti-diabetic complications, anti-Alzheimer's disease, liver and renal protection, with a complex mechanism of action involving numerous targets and signaling pathways, and scutellarin exerts these therapeutic effects mainly related to its anti-inflammatory, antioxidant, apoptosis-regulating, and vasodilating effects, but the current study was not specific enough and in-depth enough to explain the link between each target, pathway, and disease as a whole, and was unable to connect them to form a holistic mechanistic network, which requires a more in-depth study [93]. In addition, through the collection of animal or cell models in the article, it is found that most experimental results are only preliminary results, and most results lack positive control, which is a lack of reference for the clinical application of candidate drugs. Commonly used drugs, oral and intravenous injections in the formulations based on scutellarin are the mainstream of the current formulation research, among which the injectable products include breviscapine injection, Deng zhan hua Injection, breviscapine sodium chloride injection, breviscapine dextrose injection, the first two of which are more widely used in clinical applications [94].

It has been proved that scutellarin can be used in combination with other drugs to enhance therapeutic efficacy. Therefore, combining scutellarin with other drugs has a promising application prospect. Cocrystallization and nanoformulation technology occupy an important proportion in new drug development, and co-crystallization of scutellarin with other drugs for combination therapy may be a potential way of new drug development. In addition, cocrystallization and nanoformulation can also help to improve the problems of poor stability, poor water solubility, low oral bioavailability, and short in vivo half-life of scutellarin, which has a good prospect for research and development.

As a natural small molecule, scutellarin has good prospects for clinical application and is a potential therapeutic agent for tumors, atherosclerosis, cardiac and cerebral ischemic injuries, diabetic microangiopathy, hyperuricemia, and anti-Alzheimer's disease, etc. However, the current study is not deep enough, and more pharmacological mechanisms of action need to be further explored. Network pharmacology is an interdisciplinary subject between bioinformatics and pharmacology. With the breakthrough of artificial intelligence and other fields, it can better improve the efficiency of research and provide support for the mechanism exploration of candidate natural small molecules.

## Data Availability

The data supporting the findings of this study are available upon reasonable request from the corresponding author.
